# 

**Published:** 1873-01-01

**Authors:** 


### CONTENTS OF VOLUME XIV.

### A.

PAGE.

Atropia in Night-Sweats ... 22
Amaurosis Cured by Electricity ... 23
Anæsthetics ... 23
Alcoholic Stimulants, Questions in Regard to. By C. W. Earle, M.D. ... 37
American Medical Association, a Suggestion for ... 45
Amputation, Intermediate ... 47
American Medical Association ... 115, 82, 55
Alumni Association Chicago Medical College ... 55
Anasarca, Extensive Drainage in ... 54
Alcohol and Renal Diseases ... 70
Artificial Respiration, Influence of on the Circulation ... 107
American Public Health Association, Proceedings of ... 116
Ankle-joint, Excision of ... 192
Anæsthesia, Local, a New Method of Production ... 203
Anæmia and Chlorosis ... 203
Albuminous Urine, Abnormal Behavior of Under the Usual Tests ... 200

### B.

Bromide of Iron ... 204
Balsam Copaiba in Small-Pox and Scarlatina ... 142
Breech Presentation, Delivery of the Head in ... 190
Boils, their Cause and Cure ... 191
Bromide of Potassium ... 191
Belladonna in Intestinal Invagination and Hernia ... 212
British Medical Association ... 225, 223
Brain, a Case of Tumor of. By N. Bridge, M.D. ... 243
Brain, Compression of ... 267

### C.

Clavicle, Anterior Dislocation of Sternal End of ... 1
Cundurango, Experiments with. By E. Andrews, M.D. ... 13
Chloral Hydrate in Pertussis. By J. F. Stair, M.D. ... 41
Central Illinois Medical Society, Proceedings of ... 44
Cancer of the Liver, Two Cases. By M. Reece, M.D. ... 49
Cardiac Diseases, etc. By N. S. Davis, M.D. ... 52
Cabbage Leaves, Action on Ulcers and Skin Diseases ... 56
Chancre, Phagadenic, Treatment by Irrigation ... 59
Colds, Stop Them ... 71
Cæsarean Section ... 71
Chicago Society of Physicians and Surgeons ... 279, 214, 201, 187, 156, 76
Constipation in Children ... 80
Cerebro-Spinal Meningitis. By J. B. Weed, M.D. ... 121, 107
Cerebro-Spinal Meningitis. By G. D. Mitchell, M.D. ... 109
Carbazotate of Ammonia in Malarial Diseases ... 114
Cow-Pox, Report on. By J. E. Thayer, M.D. ... 123
Cerebro-Spinal Meningitis, Chronic. By N. S. Davis, M.D. ... 133
Consumption, Distribution of ... 143
Cholera. By J. S. Jewell, M.D. ... 157
Consumption, Treatment of, in the Brompton Hospital ... 166
Convulsions, Chloroform in ... 167
Carotid Artery, Ligation of ... 168
Children, the Raising of Doubled ... 168
Cerebro-Spinal Meningitis, Cases of. By J. H. Hollister, M.D. 169
Cholera, its Prevention and Treatment. Editorial ... 189
Cholera, Report on. By T. F. Worrell, M.D ... 193
Choleraic Disease, a Prophylactic and Remedy in the Preliminary Diarrhœa of ... 202
Calabar Bean ... 202
Cholera Asiatica, its Pathology and Treatment. By W. W. Wood, M.D. ... 205
Cholera, Hypodermic, Use of Belladonna and Atropia in ... 213
Cancer, its Pathology and Treatment, an Essay. By N. Senn, M.D. ... 229, 217
Croup, Membranous, a Case Relieved by Tracheotomy ... 236
Circumcision, Advantages of ... 240
Cervico-Occipital Neuralgia. By A. W. Lueck, M.D. ... 24
Continued Fever, Clinical Cases. By N. S. Davis, M.D. ... 249
Chloroform, its Administration During Sleep ... 267
Calculus Renal, on the Extraction of, more than a Century Ago ... 277

### D.

Dislocation, Three Cases. By N. Senn, M.D. ... 1
Diphtheria, Treatment of by Inhalation ... 12

PAGE.

Dissection, the Legalizing of. Editorial ... 20
Diseases of Children, Temperature in ... 67
Dysmonorrhœa ... 71
Dropsy from Disease of the Liver and Kidneys. By F. K. Bailey, M.D. ... 100
Digitalis in the Dropsies and Nephritic Diseases of Children 126
Dyspepsia, Lactic Acid in ... 143
Diarrhœa, Infantile, Zinc Oxide in ... 193
Dyspepsia, Functional, a New Method of Treatment ... 203

### E.

Electricity as a Cure for Amaurosis ... 23
Electricity as a Means of Resuscitation ... 23
Eucalyptus Globulus, Therapeutic Application of ... 23
Ether and Chloroform ... 24
Electric Bath ... 46
Eclampsia, Chloroform in ... 47
Eczema Genitale. By James N. Hyde, M.D. ... 73
Epilepsy, Brown-Sequard's Formulæ for ... 82
Endocarditis and Embolism ... 107
Eczema of the Extremities, Chronic ... 168
Exophthalmos. By F. C. Hotz ... 183
Ether, Death from Inhalation of ... 263
Ergot in Nervous Diseases ... 261
Elevations of the United States, Dictionary of ... 294
Elastic Bandage, Esmarch's Improvement on ... 298

### F.

Femur, Dislocation of ... 1
Fever, Cases of, with Destructive Parotitis. By J. F. Kelsey, M.D. ... 26
Foreign Body in the Ear, a Case of. By D. J. McMillan, M.D. 50
Fibroid Tumors of the Uterus ... 54
Franco-German War, Surgical Operations in ... 62
Fœtus, Determination of the Life or Death of ... 71
Febrile Diseases of Children, Use of Quinine in ... 94
Fistula in Ano Radical Cure of ... 222

### G.

Gastrotomy ... 66
Gelsemium in Gynæcology ... 126
Gastralgia, Pill for ... 143

### H.

Heart, First Sound of, Clinical Evidence of the Valvular Origin of the ... 11
Hæmorroids, Nitric Acid in ... 59
Heart, Functional Diseases of. By N. S. Davis, M.D. ... 78
Hypodermic Injections, the Material for ... 108
Horehound in Coughs of Children ... 126
Hard Water, Is it Wholesome? ... 142
History of Medicine. By Lyman Ware, M.D. ... 151
Hydrocele, its Causes and Nature ... 166
Hernia, Irreducible. By A. C. Olney, M.D. ... 234
Head, Gun-Shot Wound of ... 238
Hernia, Strangulated, and the Aspirator ... 255
Hypodermic Syringe, a New Form of ... 265
Hernia, on the Radical Cure of ... 278

### I.

Infant Mortality. Editorial ... 7
Inhalation in Diphtheria ... 12
Illinois State Medical Society ... 138, 104, 101
Indiana State Medical Society ... 141
Iowa State Medical Society ... 141
Invagination and Hernia, Belladonna in ... 212
Incontinence of Urine, Nocturnal, Cured by Circumcision ... 222
Intestines, Strictures of ... 253
Incisions, Studies and Experiments Regarding their Painless Production. By E. Andrews, M.D. ... 257
Intestinal Worms, a Clinic on. By Prof. N. S. Davis, M.D. ... 291

### L.

PAGE.
Lithotomy, Case of. By H. Wardner, M.D. 4
Liver, Cancer of, Two Cases. By M. Reece, M.D. 49
Locomotor Ataxy Caused by Train Movement. By G. C. Lawrence, M.D. 57
Locomotor Ataxy Treated with Nitrate of Silver 108
Lacerated Wound of Abdomen, Case of. By C. H. Norred, M.D. 114
Labor, Position in. By J. W. Smith, M.D. 145
Leucorrhœa, Carbolic Acid in 168
Lime Vapor in Membranous Croup 181
Lithotripsy, the First Operation of in America 192
Labor, Slowing of the Pulse After 212

### M.

Meningitis, Tubercular, Difficulty in Diagnosis of 21
Muscular Contraction a Cause of Fracture 60
Medical Literature, Report on 66
Mucous Diarrhœa with Pregnancy. By F. K. Bailey, M.D. 112
Morphia as a Parturient 126
Membranous Croup, Lime Vapor in. By John Bartlett, M.D. 181
Mumps, the Nature of 204
Meningitis, Albuminuria as a Symptom of 267
Nux-vomica and its Alkaloids, Abuse of 298

### N.

Night-Sweats, Atropia in 22
Northwestern Indiana Medical Society 8
Nitric Acid in Hæmorrhoids 59
National Medical Register 141
Nephrotomy 143
Narcosis from Chloroform, Treatment of by Ice in the Rectum 191
New-Born Children, Resuscitation of 215
Nux Vomica and its Alkaloids, Abuse of 298

### O.

Ovariotomy 58
Ovariotomy, Cases of. By J. F. Everett, M.D. 61
Ovariotomy, the Vaginal Operation for 284
Obstetrics and Diseases of Women and Children, Report on 88
Ohio State Medical Society 141
Ozocerite, Use of 203
Ozaena. By F. K. Bailey, M.D. 260
Otology, Report on. By S. J. Jones, M.D. 294

### P.

Prurigo and Pruritus 9
Parotitis, Destructive, with Cases of Fever 26
Parotid Gland, Diseases of in Typhus Fever 33
Pertussis, Chloral Hydrate in 41
Pericarditis, Experimental Researches in 47
Prostatic Enlargement, the Best Catheter for Patients with. By E. Andrews, M.D. 51
Peripheral Placentitis. By H. Wardner, M.D 64
Psychology in Paris 78
Poisons, Reception of During Sleep 80
Placenta Retained Three Weeks Without Putrefaction 99
Puerperal Fever 103
Pancreatic Emulsion in the Wasting Diseases of Children 104
Puerperal Diseases, Septicæmia, etc. 105
Progressive Muscular Atrophy 114
Placenta Praevia, a Fatal Case. By A. B. McCandless, M.D. 135
Psoriasis, Treatment of 167
Pessaries, the Employment of 167
Phosphorus, Value of in Nervous Diseases 191
Pulmonary Phthisis, its Curability 241
Pericardium, Puncture of, by Subcutaneous Aspiration 239
Polypus Uteri. By F. K. Bailey, M.D. 259
Poisons, Organic. By E. W. Gray, M.D. 271
Pneumonia, Double, Clinical Case. By N. S. Davis, M.D. 275
Pleurisy, Acute, Clinical Case. By N. S. Davis, M.D. 276
Puerperal Convulsions, Clinical Lectures on 280
Poisons, the Doctrine of 285

### Q.

Quinia, Oxytoxic, Action of 47
Quinia in Whooping-Cough 107

### R.

PAGE.
Rubeola. By N. S. Davis, M.D. 42
Resuscitation in Newly-Born Children 59
Renal Diseases and Alcohol 70
Rheumatism, Cases. By F. K. Bailey, M.D. 85
Rheumatic Bronchitis, Cases of. By N. S. Davis, M.D. 136
Rock River Medical Society 141
Rheumatic Carditis, a Case of Suddenly Fatal 175
Rectal Sound, a New Form of. By E. Andrews, M.D. 244
Report of Surgeon-General U. S. Army for 1873 293

### S.

Scarlatina, Renal Dropsy Following. By N. S. Davis, M. D. 28
Smells, Relation of to Disease 22
Small-Pox, Abortive Treatment of. By W. E. Bessey, M.D. 33
Septicæmia 47
Syphilis, Treatment of in the Marine Hospital 57
Sterility from Narrow Os Uteri 58
Scarlatinal Dropsy, with Suppression of Urine, and Convulsions. By E. S. Cleveland, M.D. 65
Sewerage Question 72
Stricture, Impassable, New Method of Treating 80
Starch, Digestion of in Infancy 107
Shoulder Presentation, a Case of. By G. D. Mitchell, M.D. 111
Spleen, Action of Cold Water on 142
Sulphate of Iron in Suppuration 142
Spina Bifida 143
Superior Maxillary, Excision of 200, 168
Sporadic Cholera, Two Cases. By F. H. Davis, M.D. 185
Sciatica, Report of a Case of. By T. D. Washburn, M.D. 196
Skin Diseases. By Hood 199
Strictures of Urethra and Rectum. By W. C. Lyman, M.D. 224
Subcutaneous Injections 236
Skull, Comminuted Fracture of. By Prof. E. Powell 264
Scarlatinal Pleurisy, Thoracentesis in 268
Scientific Congress at Rome, The 293

### T.

Temperature in Diseases of Children 68
Tuberculosis, Effects of Pregnancy on 93
Thermometry of Yellow Fever 106
Transplantation of a Rabbit's Cuticle to the Human Eye Successfully Performed 167
Tetanus, Chronic, Treated by Excision of the Cicatrix 212
Typhoid Fever, Spread of in London, by Milk Supply 237
Thorasic Abscess. By J. Schneck, M.D. 245
Thoracentesis in Scarlatinal Pleurisy 268
Tracheotomy, a Successful Case 236

### U.

Unusual Cases. By Ira Manly, M.D. 25
Uterus, Fibroid Tumors of, After Treatment of 59, 54
Uterine Cancer, Conservative Purpose of 59
Uræmia 191
Uterus and Ovaries, Absence of in Three Sisters 227
Uterine Inflammation, on the Prevention of 282

### V.

Varicose Veins 12
Vaginal Obstruction, Case of. By Ira Manly, M.D. 25
Vesico Vaginal Fistulæ, a Case of. By Mary H. Thompson. 85
Variola and Typhus Fever in Same Individual at Some Time 96
Varicose Veins, a Case of. By D. T. Nelson, M.D. 97
Ventilation and Warming 106
Vermifuge Syrup 143
Varicocele, its Easy, Safe, and Radical Cure 241
Vaccinal Syphilis 253

### W.

Whooping-Cough, Treatment of 192

### Z.

Zinc Ozide in the Diarrhœa of Infants 192

Zinc Ozide in the Diarrhœa of Infants 224

Money Receipts.—We acknowledge remittances received from Drs. J. M. Barlow, J. O. Curtis, W. W. Grout, J. B. Reed, J. C. Allaben, J. Schneck, A. H. Smith, J. C. Adrian, Jos. Culbertson, G. E. Bowers, A. P. Nelson, E. R. Atwood, M. P. Hatfield, J. R. Eversole, and L. D. Humphrey.

